# First-in-Human Intramediastinal Taurolidine Irrigation for *Candida albicans* Mediastinitis After Biological Bentall Procedure

**DOI:** 10.3390/jcdd13050204

**Published:** 2026-05-12

**Authors:** Ziyad Gunga, Augustin Rigollot, Agnès Godat, Lars Niclauss, Matthias Kirsch

**Affiliations:** Department of Cardiovascular Surgery, Lausanne University Hospital (Centre Hospitalier Universitaire Vaudois (CHUV)), 1011 Lausanne, Switzerlandagnes.godat@chuv.ch (A.G.); lars.niclauss@chuv.ch (L.N.); matthias.kirsch@chuv.ch (M.K.)

**Keywords:** mediastinitis, taurolidine, *Candida albicans*, novel procedure, fungal infection, cardiac surgery

## Abstract

Background: Post-sternotomy mediastinitis remains a devastating complication of cardiac surgery. Although most cases are bacterial, fungal mediastinitis due to *Candida albicans* is rare, aggressive, and particularly difficult to treat because of biofilm formation, prosthetic involvement, and limited penetration of systemic antifungal agents into infected tissues. Taurolidine is a taurine-derived antimicrobial compound with broad antibacterial, antifungal, and anti-biofilm properties that has shown promising results in catheter-related infection prevention and cardiac implantable electronic device surgery. Case summary: We report, to our knowledge, the first intramediastinal use of taurolidine for *Candida albicans* mediastinitis after biological Bentall surgery. Following urgent resternotomy and extensive debridement, 200 mL of taurolidine solution was instilled into the mediastinum for 60 min, then aspirated. Postoperatively, taurolidine irrigation via mediastinal drainage was combined with negative-pressure wound therapy and systemic antifungal treatment. Results: Rapid microbiological sterilization was achieved, inflammatory markers normalized, and follow-up computed tomography demonstrated complete resolution of mediastinal infection. Delayed sternal closure was then performed successfully without recurrence at 6-month follow up. Conclusion: To our knowledge, this represents the first reported use of intramediastinal taurolidine irrigation for fungal mediastinitis following cardiac surgery. Intramediastinal taurolidine irrigation may represent a promising adjunctive strategy for mediastinitis after cardiac surgery in high-risk patients. Further clinical evaluation is warranted.

## 1. Introduction

Deep sternal wound infection and mediastinitis remain among the most serious complications after cardiac surgery, with reported incidence generally ranging from approximately 0.5% to 2% after median sternotomy and with substantial associated morbidity, prolonged hospitalization, and mortality [1,2,3,4]. While most mediastinal infections are bacterial, fungal mediastinitis is increasingly recognized in critically ill or immunocompromised patients and in those requiring complex reconstructive procedures involving foreign material [2]. Among fungal organisms, *Candida albicans* is the most commonly encountered pathogen [3].

Fungal mediastinitis is particularly challenging because infection often develops in poorly vascularized tissues, around prosthetic surfaces, and within inflammatory or necrotic cavities, where systemic therapy alone may be insufficient. In addition, Candida species readily form biofilms on prosthetic material and devitalized tissue, promoting persistence of infection and reduced susceptibility to antifungal therapy. Consequently, Candida mediastinitis remains associated with a reported mortality of approximately 40–60% [3]. These characteristics often necessitate a multimodal strategy combining debridement, prolonged systemic antifungal therapy, repeated local control, and delayed reconstruction.

Local mediastinal irrigation has historically been explored using saline, povidone-iodine, and antibiotic-containing solutions. However, evidence specific to the post-sternotomy mediastinum remains limited, and contemporary guidance emphasizes surgical debridement, systemic antimicrobial therapy, and negative-pressure wound therapy rather than reliance on any single irrigant [4,5,6]. American Association for Thoracic Surgery (AATS) guidance specifically advises against dilute povidone-iodine mediastinal irrigation in deep sternal wound infection with mediastinitis [4,5,6].

Taurolidine (TauroPharm Gmbh, Waldbuttelbrunn, Germany) is a taurine-derived antimicrobial agent with broad activity against Gram-positive bacteria, Gram-negative bacteria, and fungi, including Candida species [7,8,9]. Its mechanism differs from that of conventional antibiotics, acting through reactive methylol groups that damage microbial cell wall structures and neutralize bacterial toxins [8,9]. Taurolidine has also demonstrated anti-biofilm properties and has been used in catheter-related infection prevention and, more recently, in cardiac implantable electronic device procedures [10,11,12,13,14]. These properties make it an appealing candidate for infections involving foreign material and contaminated low-flow spaces. We therefore report, to our knowledge, the first intramediastinal application of taurolidine irrigation for *Candida albicans* mediastinitis after biological Bentall surgery.

## 2. Case Presentation

**Figure 1:**
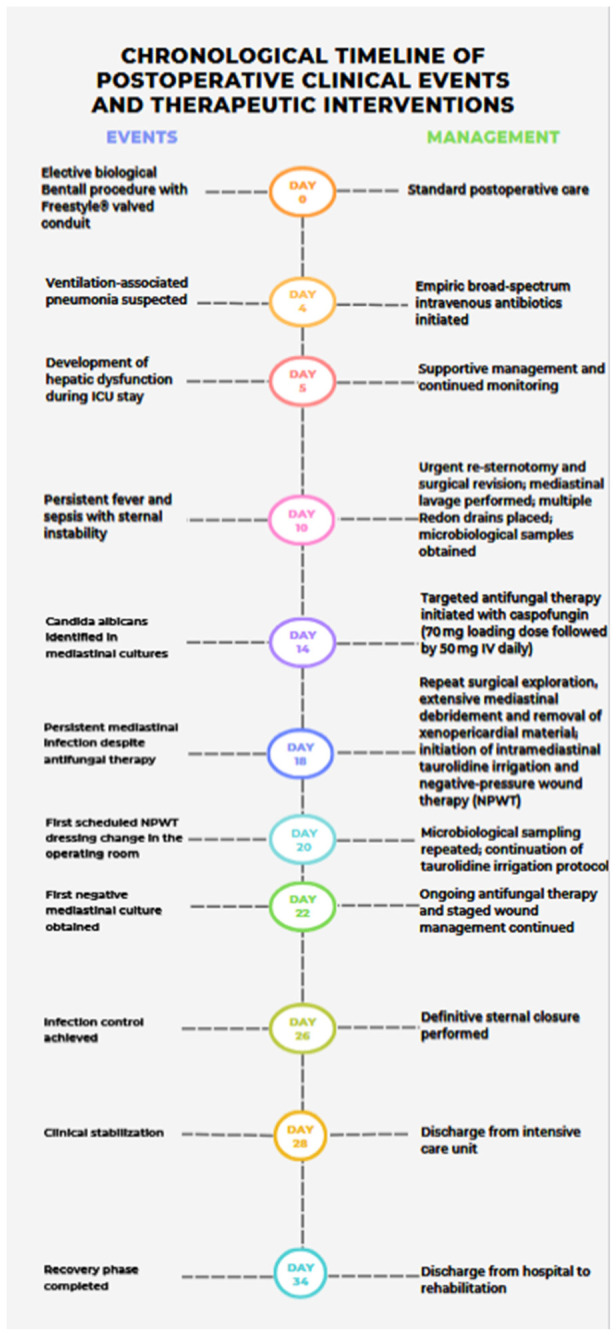
Chronological timeline of postoperative clinical events and therapeutic interventions.

A 56-year-old man with an ascending aortic aneurysm and severe aortic valve insufficiency underwent elective aortic root replacement using a Freestyle^®^ biological valved conduit (Medtronic, Irvine, CA, USA) (Figure 1). The immediate postoperative course was complicated by hepatic dysfunction and ventilation-acquired pneumonia, resulting in a prolonged stay in the intensive care unit. Management required endotracheal intubation and empiric broad-spectrum intravenous antibiotic therapy, with subsequent supportive care. On postoperative day ten, the patient developed fever, erythema of the sternal wound, and sternal instability, accompanied by rising inflammatory markers. Contrast-enhanced computed tomography demonstrated an anterior mediastinal fluid collection with surrounding inflammatory changes, highly suggestive of mediastinitis (Supplementary Materials, Figure S1). Urgent re-sternotomy was therefore performed and revealed a retrosternal purulent collection consistent with deep sternal wound infection. After obtaining multiple microbiological samples, a thorough mediastinal lavage with povidone-iodine solution was performed. Multiple mediastinal Redon drainage catheters (B. Braun Medical SA, Sempach, Switzerland) were subsequently positioned within the retrosternal space to ensure continuous postoperative drainage.

Intraoperative cultures subsequently grew *Candida albicans*, and targeted systemic antifungal therapy with Caspofungin was initiated in accordance with susceptibility testing and infectious-disease recommendations. Treatment consisted of a 70 mg intravenous loading dose followed by a maintenance dose of 50 mg intravenously once daily for a duration of 6 weeks. The other antibiotics were stopped. Despite this management, mediastinal drains continued to yield purulent output, and inflammatory markers remained elevated. The patient remained febrile and tachycardic, although without signs of septic shock, and blood cultures remained negative for candidemia. Given the persistence of infection, a decision was made to return to the operating room and revise the treatment strategy.

**Figure 2:**
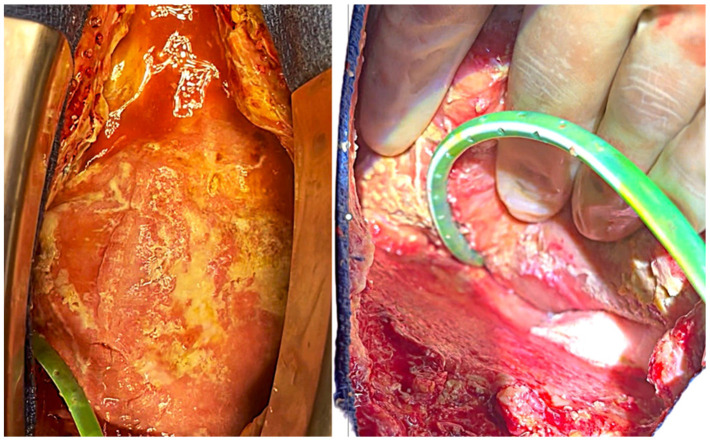
Intraoperative findings showing multiple white plaque-like deposits on the epicardial surface, involving both the anterior (**left**) and posterior (**right**) aspects of the heart. The macroscopic appearance was highly suggestive of fungal colonization, later confirmed by microbiological cultures as *Candida albicans*.

Repeat intraoperative exploration revealed diffuse inflammatory mediastinal tissue and multiple white plaque-like deposits over the epicardium and diaphragmatic pericardium, macroscopically suggestive of fungal infection (Figure 2). Extensive surgical debridement was performed, and previously implanted xenopericardial material, for pericardial approximation, was removed. Repeat intraoperative cultures again confirmed *Candida albicans*, while blood cultures remained negative. Careful intraoperative inspection of the Freestyle^®^ bioprosthetic conduit revealed no macroscopic evidence of prosthetic infection, structural involvement, or pseudoaneurysm formation.

## 3. Taurolidine Irrigation Protocol

Following aggressive surgical debridement, 200 mL of taurolidine solution (TauroPace^®^) (TauroPharm Gmbh, Waldbuttelbrunn, Germany) was instilled into the mediastinum and left in situ for 60 min before aspiration. Postoperatively, negative pressure wound therapy (NPWT) was applied, and repeat taurolidine instillation was performed 24 h later via a mediastinal drain using the same volume and dwell time prior to activation of the vacuum system. Dressing changes and wound reassessment were performed every 48 h (see Table 1).

At each re-exploration and dressing change, systematic microbiological sampling was obtained from the intrapericardial space, sternal edges, and superficial wound layers. Systemic antifungal treatment continued without modification according to institutional infectious disease recommendations and pathogen susceptibility testing.

## 4. Results

Clinical improvement occurred rapidly after initiation of the combined treatment strategy. Inflammatory markers progressively declined and normalized after one week of therapy, while repeat microbiological cultures became sterile within 48 h of the initial debridement and taurolidine treatment. Follow-up computed tomography demonstrated complete resolution of mediastinal inflammation, with no residual abscess or persistent fluid collection. Clinically, no recurrence of the previously observed patchy white fungal lesions was noted.

**Figure 3:**
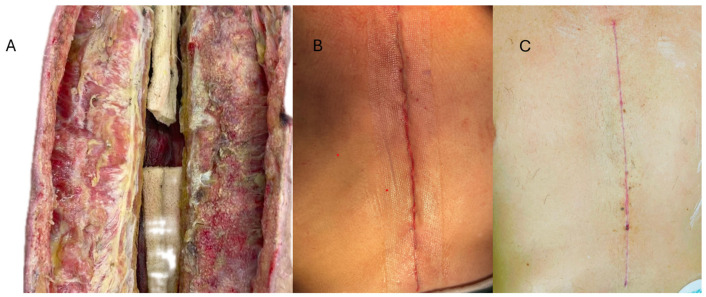
Stepwise management from extensive mediastinal debridement and sternal edge freshening with NPWT (**A**) to definitive sternal and wound closure using STRATAFIX™ barbed sutures and DERMABOND™ PRINEO™ (**B**). The final postoperative appearance with uneventful healing after 8 weeks (**C**).

Once infection control had been achieved, delayed sternal closure was performed after a final intraoperative taurolidine rinse, including irrigation over the newly placed stainless-steel sternal wires. The presternal fascia and subcutaneous tissue were reapproximated using triclosan-coated STRATAFIX barbed sutures™ (Johnson & Johnson MedTech, Raritan, NJ, USA), chosen to provide secure, evenly distributed tension and tight tissue apposition. To further support surgical-site infection prevention after multiple re-entries, the skin was closed with the DERMABOND™ PRINEO™ skin-closure system™ (Johnson & Johnson MedTech, Raritan, NJ, USA) as the final protective layer, in line with the favorable previously reported experience in cardiac surgery by Gunga et al. [15] (Figure 3). The patient was discharged from the intensive care unit after four weeks and left the hospital one week later for rehabilitation. Subsequent recovery was uneventful, and no recurrence of infection was observed during a 6-month follow-up (see Figure 1).

## 5. Discussion

This case describes, to our knowledge, the first reported intramediastinal use of taurolidine following cardiac surgery. The clinical interest of this approach lies in the convergence of several difficult features: a deep post-sternotomy infection, involvement of prosthetic material, devascularized mediastinal planes, and a fungal organism known for biofilm formation and therapeutic persistence [2,3].

Several mediastinal irrigation solutions have previously been described for post-sternotomy mediastinitis or deep sternal wound infection, including normal saline, povidone-iodine, antibiotic-containing lavage solutions, and, more rarely, superoxidized or other antiseptic preparations. However, the evidence base for cardiac mediastinal irrigation specifically is remarkably limited. A systematic review and meta-analysis of randomized controlled trials found that although aqueous povidone-iodine irrigation may reduce surgical-site infection in general surgery, the single cardiac mediastinal study included showed no benefit over saline, while antibiotic irrigation did not demonstrate a consistent advantage [5]. A 2024 network meta-analysis likewise found that antiseptic incisional wound irrigation reduces surgical-site infection in general surgery, whereas antibiotic wound irrigation should be avoided because of weaker certainty of benefit and antimicrobial-resistance concerns [16]. These broader findings are informative, but they should not be directly over-extrapolated to the post-sternotomy mediastinum.

In current cardiac practice, no irrigant can therefore be considered definitively superior based on high-level evidence. Contemporary guidance instead emphasizes early and thorough debridement, culture-directed systemic antimicrobial therapy, and negative-pressure wound therapy when delayed closure is required [4,6,17]. The role of local irrigation remains adjunctive rather than central. Indeed, AATS guidance advises against dilute povidone-iodine irrigation in deep sternal wound infection with mediastinitis [4,6].

An additional potential advantage of taurolidine in this setting is that improved early local infection control may, in selected cases, help avoid escalation to more invasive salvage procedures. In severe post-sternotomy mediastinitis, particularly when infection involves exposed prosthetic material such as Dacron grafts or reconstructed xenopericardial surfaces, definitive management may require radical debridement followed by flap-based reconstruction, including pectoralis advancement or omental transposition, to obliterate dead space and wrap or cover infected mediastinal structures [17,18]. These approaches remain valuable and sometimes indispensable, but they are inherently invasive and may require additional abdominal or complex reconstructive surgery. Contemporary expert consensus and surgical series continue to recognize omental and muscle flaps as established options for complex mediastinitis and thoracic prosthetic graft infection, particularly when there is major tissue loss, persistent infection, or the need to salvage exposed vascular graft material [17,18,19,20]. In this context, a local adjunct such as taurolidine is attractive not because it replaces flap surgery when flap coverage is clearly indicated, but because it may contribute to earlier reduction in microbial burden, biofilm persistence, and infected debris, thereby potentially limiting progression to scenarios requiring extensive mediastinal reconstruction, prosthetic wrapping, or omentoplasty.

Within this landscape, taurolidine is biologically attractive. It has broad antimicrobial and antifungal activity, interferes with microbial adhesion and biofilm persistence, and can neutralize bacterial endotoxins and exotoxins [7,8,9]. These properties are especially relevant in the mediastinum, where persistent contamination may be sustained by necrotic debris, exudate, prosthetic surfaces, and poorly perfused tissue planes. In that context, a useful irrigant should ideally do more than provide mechanical lavage: it should also help reduce microbial burden, destabilize biofilm, and facilitate clearance of inflammatory debris after debridement. Taurolidine has demonstrated a favorable safety profile in multiple clinical settings, including catheter-related infection prevention and cardiac implantable electronic device surgery, with minimal systemic toxicity reported.

The strongest clinical experience with taurolidine comes from other domains. Taurolidine lock solutions have been shown in randomized studies and meta-analyses to reduce catheter-related bloodstream infections [10,11]: for example, Bisseling et al. showed an infection-free survival of 641 days with taurolidine, against 175 days for heparin control. More recently, observational cardiothoracic data have suggested favorable results for taurolidine-containing solutions in cardiac implantable electronic device surgery and related surgical fields [12,14]. These settings differ from fungal mediastinitis after sternotomy, but they share several mechanistic features, notably the presence of foreign material, confined operative spaces, and biofilm-prone surfaces. This makes cautious extrapolation biologically plausible, even though direct evidence for mediastinal use remains absent.

Beyond its role as a rescue adjunct in established mediastinal infection, taurolidine may also deserve prospective evaluation within an ERAS-based infection-prevention bundle for cardiac surgery. More recently, the Enhanced Recovery After Surgery (ERAS) Cardiac Society proposed a dedicated turnkey order set for surgical-site infection prevention, underscoring that benefit derives from reliable implementation of a reproducible care pathway across the preoperative, intraoperative, and postoperative phases rather than from any isolated intervention [21,22]. Within such a framework, taurolidine could be conceptualized as a local adjunctive irrigating agent integrated into a broader wound- and mediastinal-protection strategy, particularly in selected high-risk settings such as redo sternotomy, prolonged operative time, obesity, diabetes, re-exploration for bleeding, prosthetic or biologic material implantation, and prolonged intensive care exposure. Given its broad antimicrobial and antifungal activity, anti-biofilm effects, and low apparent propensity for resistance selection, taurolidine may offer a biologically plausible means of reducing local microbial burden and assisting clearance of debris at the time of closure or re-exploration [7,8,9,12,14]. However, such an application remains investigational and should presently be framed as a hypothesis-generating ERAS-compatible innovation, to be studied prospectively in conjunction with standardized bundle adherence, rather than as a standalone preventive measure.

In the present case, taurolidine was administered as part of a multimodal treatment strategy including repeated surgical debridement, removal of infected xenopericardial material, systemic antifungal therapy, and negative-pressure wound therapy. The favorable outcome cannot, therefore, be attributed to taurolidine alone. Nevertheless, the rapid microbiological clearance, normalization of inflammatory markers, radiologic resolution of mediastinal inflammation, and successful delayed sternal closure support the feasibility and safety of this adjunctive intramediastinal approach. A total of eight taurolidine 200 mL irrigations were performed during staged management, including a final instillation at the time of definitive sternal closure. Taurolidine may be particularly relevant in fungal mediastinitis. Compared with bacterial mediastinitis, fungal infections are often harder to eradicate, more likely to involve biofilm, and more frequently associated with prolonged critical illness and prosthetic contamination [3]. A local irrigant with antifungal and anti-biofilm properties is therefore conceptually appealing. In addition, its use may help decrease the local infectious burden while facilitating the removal of debris and exudate that perpetuate inflammation and microbial persistence.

This report has clear limitations. It concerns a single patient and cannot establish efficacy, superiority, or optimal dosing. The observed success may reflect the combined effect of surgery, systemic antifungal therapy, wound management, and host factors rather than taurolidine per se. In addition, published clinical evidence for taurolidine in cardiothoracic surgery currently remains strongest in device-related applications and preventive settings, not in established mediastinal fungal infection [12,13,14]. Accordingly, taurolidine should presently be regarded as a promising adjunct rather than an evidence-based standard.

Future work should explore its role in fungal mediastinitis, polymicrobial deep sternal wound infection, prosthetic-associated thoracic infection, and high-risk redo sternotomy. Prospective registries and carefully designed multicenter studies will be needed to define indications, safety, volume, dwell time, and integration with negative pressure wound therapy and staged closure.

## 6. Conclusions

This report describes the first intramediastinal application of taurolidine following cardiac surgery. In combination with surgical debridement, systemic antifungal therapy, and negative pressure wound therapy, taurolidine irrigation was associated with rapid infection control and successfully delayed sternal closure. Owing to its antimicrobial, antifungal, and anti-biofilm properties, taurolidine may represent a promising adjunctive strategy for complex mediastinal infections. It may also warrant prospective evaluation as a local adjunct within Enhanced Recovery After Surgery (ERAS) surgical-site infection prevention bundles for selected high-risk cardiac surgical patients. Further clinical investigation is needed to define its role in mediastinal infection management.

## Figures and Tables

**Table 1:** Intramediastinal Taurolidine irrigation Protocol during staged management of fungal mediastinitis.

Step	Timing	Intervention	Details
1	Day 0: Surgical re-exploration	Debridement and initial irrigation	Extensive mediastinal debridement followed by warm saline irrigation and microbiological sampling
		Initial taurolidine instillation	200 mL taurolidine (TauroPace^®^) instilled intramediastinally
		NPWT initiation	Placement of white and black NPWT sponges; continuous suction −125 mmHg
2	Day 1: Bedside management	Taurolidine instillation via mediastinal drain	NPWT paused; 200 mL taurolidine instilled via drain, which was temporarily clamped
		Dwell time and NPWT reactivation	60-min dwell time, then NPWT restarted at −125 mmHg
3	Day 2: Operating room re-exploration	Dressing change and inspection	NPWT dressing removed; mediastinum inspected
		Microbiological sampling	Tissue cultures obtained from intrapericardial space, sternal edges, and superficial layers
		Taurolidine irrigation and NPWT reapplication	200 mL taurolidine instilled after saline irrigation; NPWT dressing replaced
4	Day 3: Bedside management	Repeat drain-based instillation	NPWT paused; 200 mL taurolidine instilled via mediastinal drain
		Dwell time and NPWT reactivation	60-min dwell time, NPWT restarted at −125 mmHg
5	Ongoing management	Staged wound care	NPWT dressing changes every 48 h with repeat microbiological sampling, until closure is possible
6	Final surgery	Definitive sternal closure	Final 200 mL taurolidine irrigation prior to closure; sternum and skin closed using STRATAFIX™ barbed sutures and DERMABOND™ PRINEO™ system

## Data Availability

The original contributions presented in this study are included in the article and Supplementary Materials. Further inquiries may be directed to the corresponding author.

## Supplementary Materials

The following supporting information can be downloaded at: https://www.mdpi.com/article/10.3390/jcdd13050204/s1, Figure S1: Contrast-enhanced chest CT showing a persistent post-operative pericardial/anterior mediastinal fluid collection despite drains in place. The sagittal re-construction highlights a retrosternal collection extending between the sternum and the anterior cardiac surface.
