# Supplementary material for: First-in-Human Intramediastinal Taurolidine Irrigation for Candida albicans Mediastinitis After Biological Bentall Procedure
Source: J Cardiovasc Dev Dis. 2026 May 12;13(5):204. doi: 10.3390/jcdd13050204 (PMC13207969; doi:10.3390/jcdd13050204)
Supplement: Supplementary file 1 [file jcdd-13-00204-s001.zip › jcdd-4230939-supplementary.pdf]

Supplementary Materials

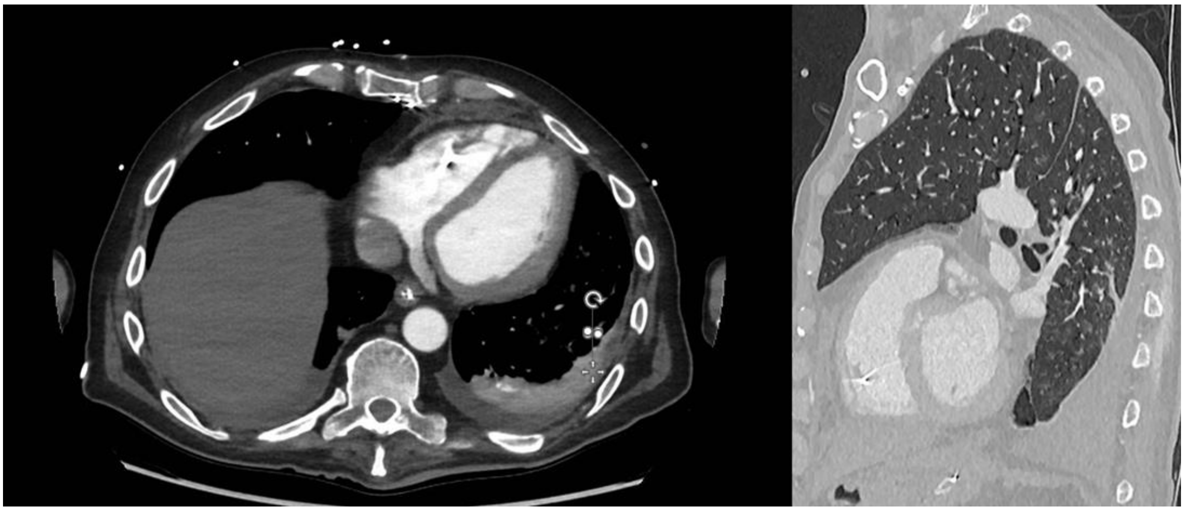

Figure S1. Contrast-enhanced chest CT showing a persistent post-operative pericardial/anterior mediastinal fluid collection despite drains in place. The sagittal re-construction highlights a retrosternal collection extending between the sternum and the anterior cardiac surface.
